# Sustainable Sulfur-rich Copolymer/Graphene Composite as Lithium-Sulfur Battery Cathode with Excellent Electrochemical Performance

**DOI:** 10.1038/srep25207

**Published:** 2016-04-28

**Authors:** Arnab Ghosh, Swapnil Shukla, Gaganpreet Singh Khosla, Bimlesh Lochab, Sagar Mitra

**Affiliations:** 1 Electrochemical Energy Storage Laboratory, Department of Energy Science and Engineering, Indian Institute of Technology, Bombay, Powai, Mumbai 400076, INDIA; 2 Department of Chemistry, School of Natural Sciences, Shiv Nadar University, Gautam Budh Nagar, Uttar Pradesh 203207, INDIA

## Abstract

A sulfur-rich copolymer, poly(S-r-C-a) has been synthesized via a sustainable route, showing the utility of two major industrial wastes- elemental sulfur (petroleum waste) and cardanol (agro waste), to explore its potential as cathode material for Li-S batteries. The sulfur-rich copolymer exhibited a reduction in the active material dissolution into the electrolyte and a low self-discharge rate behavior during the rest time compared to an elemental sulfur cathode, indicating the chemical confinement of sulfur units. The presence of organosulfur moieties in copolymer suppress the irreversible deposition of end-discharge products on electrode surfaces and thus improve the electrochemical performances of Li-S batteries. This sulfur copolymer offered a reversible capacity of 892 mA h g^−1^ at 2nd cycle and maintained the capacity of 528 mA h g^−1^ after 50 cycles at 200 mA g^−1^. Reduced graphene oxide (rGO) prepared via a sustainable route was used as a conductive filler to extract the better electrochemical performances from this sulfur copolymer. Such sustainable origin batteries prepared via economically viable showed an improved specific capacity of ~975 mA h g^−1^ after 100 cycles at 200 mA g^−1^ current rate with capacity fading of 0.15% per cycle and maintained a stable performance over 500 cycles at 2000 mA g^−1^.

In the recent past, the problem of rapidly depleting energy sources has shifted the outlook towards the exploration of high performing energy storage devices sourced from sustainable routes. In this context Li-S batteries have come up as a promising alternative exhibiting wide applicability ranging from electronic devices to electrical vehicles. Elemental sulfur (S) bestow several attractive inherent advantages mainly high theoretical energy density (2600 Wh kg^−1^) and capacity (1672 mA h g^−1^), economic viability and environmental benignity to infer its utility as cathodic material^1^,^2^,^3^.

Despite such attractive properties, certain drawbacks mainly the leachability of sulfur from electrode and high solubility of intermediate discharged products (higher-order polysulfides) into electrolyte solvent have impeded its usage for further commercialization in Li-S batteries. The dissolution of sulfur from electrode results low active material utilization which further leads to poor cycling stability and rapid capacity fading. On the other hand, the dissolved intermediate higher-order polysulfides migrate towards the anode, chemically react with lithium metal and reduce to insoluble/insulator lower order lithium polysulfides (Li_2_S_3_/Li_2_S_2_). These insulating lower order polysulfides then irreversibly deposit on lithium anode surface accounted to hamper Li^+^ ion transport. However, severe shuttle behavior in Li-S batteries could obstruct the ionic transport which leads to device failure^4^. Physical encapsulation of active sulfur into various porous host materials by melt-diffusion strategies^5^,^6^,^7^,^8^ has been widely followed till the date to resolve the dissolution issues. For example, Nazar *et al*. described physical encapsulation of sulfur by highly ordered mesoporous carbon CMK-3^5^. This highly ordered nanostructured carbon/sulfur composite cathode exhibited a capacity of about 1000 mA h g^−1^ for initial 20 cycles. Similarly, a sulfur-TiO_2_ yolk-shell nanocomposite with physically confined sulfur exhibited an initial specific capacity of 1030 mA h g^−1^ at 836 mA g^−1^, with a high coulombic efficiency of 98.4% and an excellent capacity retention of 67% after 1000 cycles, as reported by Cui *et al*.^9^ Wang *et al*. reported a composite with sulfur embedded in polyacrylonitrile delivered a specific capacity of 850 mA h g^−1^ in the first cycle and 600 mA h g^−1^ after 50 cycles^10^. These additives can physically confined the sulfur molecules and its discharge products within the void spaces and thereby improving the active material utilization as well as life time of Li-S batteries. However, as mentioned by Zhou *et al*.^11^, the possibility to leach out of physically bound sulfur from its reserviors still persists. Sulfurized carbon is another strategy for designing cathode materials in which short sulfur chains are covalently bound onto the surface of carbon particles^12^. Yan *et al*. immobilized sulfur (68 wt%) on the surfaces of functionalized carbon nanotubes (CNTs) at 300 °C and the electrode showed a good performance, 1180 mA h g^−1^ of sulfur over 300 cycles at 0.25 C, 799 mA h g^−1^ over 600 cycles at 0.75 C, 810 mA h g^−1^ over 500 cycles at 1 C, and 400 mA h g^−1^ over 500 cycles at 2C^13^. Amongst conducting polymers such as polythiophene, polyaniline (PANI), polyacrylonitrile (PAN) etc. has been explored for binding the sulfur moiety. Physical encapsulation via core/shell architecture of sulfur/polythiophene composite showed a high initial discharge capacity of 1119 mA h g^−1^ and retained 69.5% of the capacity after 80 cycles which was accounted to the polysulfide dissolution^14^. A high temperature mediated chemically bonded sulfur with PAN at 450 °C showed an initial specific capacity of 523 mA h g^−1^ and the capacity retention remained about 90% after 380 cycles^15^. A self-assembled sulfurized PANI-nanotube (SPANI-NT)/S molecular composite was synthesized via *in-situ* vulcanization process at 280 °C. This composite showed a discharge capacity of 837 mA h g^−1^ after 100 cycles at a 0.1 C rate^16^. Sulfurized PANI (SPANI), synthesized via wet chemical method, which involved *in-situ* chlorination and vulcanization reactions, showed stable reversible capacities of ~720, ~610 and ~510 mA h g^−1^ at 0.3, 0.6 and 1 C, respectively over 200 cycles^17^. However, excepting a few cases^18^,^19^, the sulfur loading reported in aforementioned strategies are limited to only ~60–70%, accounting for low energy density Li-S batteries affecting their practical utility. Moreover, these stategies followed some advanced, expensive and unsustainable synthetic procedures and hence unamenbale to large scale production. Therefore, challenges still persist in the large scale production of such sulfur based cathode materials with high sulfur loading using affordable and sustainble routes.

With an aim to alleviate the aforementioned challenges in an inexpensive, sustainable manner researchers have explored the chemical confinement of sulfur^20^,^21^,^22^. Recently Pyun *et al*. pioneered the synthetic procedure of copolymerization of elemental sulfur with 1,3-diisopropenylbenzene (DIB) using inverse vulcanization which allowed incorporation of large percentages (90–99%) of elemental sulfur into the sulfur copolymer^21^. The inverse vulcanization strategy showed tremendous potential towards the development of inexpensive, facile and scalable methodology for the preparation of high performance Li-S batteries. Gao *et al*. also utilized the DIB monomer followed by thiol-ene and Menschutkin quaternization reactions demonstrating solution processible sulfur copolymers as cathode-active materials for Li-S batteries^23^. Afterwards, several petro-origin organic monomers, such as namely 1,3-diethynylbenzene^24^, 1,4-diphenylbutadiyne^25^ etc. have been explored in such direction. However, the depleting availability and toxic nature of petro-effluents can hindered the large scale applications of such sulfur-rich copolymers. Therefore, parallel to the inexpensive, scalable and facile route, a sustainable as well as green approach is also expected for synthesizing sulfur-rich polymeric cathode materials.

Other major shortcomings associated with sulfur based cathodes include poor electronic conductivity of sulfur (~10^−30^ S cm^−1^) and consequent low utilization of active materials^26^. In order to counter the electrical conductivity issue^27^,^28^, incorporation of conductive carbon additives such as commercial carbon black^29^, carbon nanotubes^30^, ultrathin graphite sheets^31^, mesoporous carbon^32^, active carbon^33^, graphene oxide (GO)^34^, graphene^35^ etc. have been extensively explored. Among the aforestated conductive carbon additives, both graphene oxide (GO) and reduced graphene oxide (rGO) are most-often used as ideal conductive and flexible additives to increase electronic/ionic conductivity as well as to reduce the pulverization of active sulfur, thanks to their good electronic conductivity^36^, high surface area, high flexibility, broad electrochemical window etc^37^. In addition, both GO and rGO have shown higher ability to retain lithium polysulfides owing to presence of oxygen containing functional groups on their surface and oxophilic nature of lithium polysulfides^38^. In comparison to physical methods for preparation of graphene, chemical reduction of graphene oxide has received widespread attention amongst others due to favourable economic implications and ease of synthesis^39^. However, the chemically reducing agents namely, sodium borohydride, hydrazine hydroquinone etc^40^,^41^,^42^,^43^. are toxic and explosive in nature which has hindered the large applications of rGO at commercial scale. Furthermore, the chemically reduced graphene oxide using these reducing agents tends to aggregate because of π-π stacking^44^. Therefore, an alternative, safer and green approach is required to synthesize rGO.

In this work, at first in order to explore a “sustainable electrode material”^45^ we have synthesized a copolymer derived from cardanol based benzoxazine with high loading of sulfur (90% by wt.; abbreviated as S90) and used as cathode material in Li-S battery. Cardanol apart from being a renewable raw material^46^ is responsible for the molecular tailoring of the benzoxazine (C-a) in a manner which eases the melt polymerization with sulfur. More so, high thermal stability eradicate the possibility of monomer degradation at the high temperatures required for copolymerization^47^,^48^. Benzoxazine moiety in itself offers a multitude of reaction sites which facilitates the chemical incorporation of sulfur in such high percentages. Owing to the chemical attachment of sulfur with the organic part in sulfur copolymer, active material dissolution into the electrolyte was found to reduce and thus improve the Li-S battery performances. Due to the polymeric nature and presence of two insulating components- sulfur and C-a (organic monomer), the copolymer showed very low conductivity resulting to a poor electrochemical performances in Li-S batteries. Therefore, a conductive composite of the copolymer was synthesized using reduced graphene oxide (rGO), derived by chemical reduction of GO via naturally occurring phenolic compound, gallic acid^49^. Gallic acid is a cheap, naturally occurring and environmentally friendly antioxidant. This natural product has already been used to reduce graphene oxide (GO). The reduced GO so obtained exhibited high surface area as well as good dispersion in organic solvents because of the stabilizing effect of gallic acid^49^. This allowed a uniform dispersion of reduced graphene oxide throughout the sulfur copolymer, resulting to the excellent electrochemical performances. For comparison, reduced graphene oxide was also synthesized via a non-sustainable hydrazine route (abbreviated as rGO_(NS)_) and used as conductive additive in S90 copolymer cathode.

## Results and Discussion

**Figure 1:**
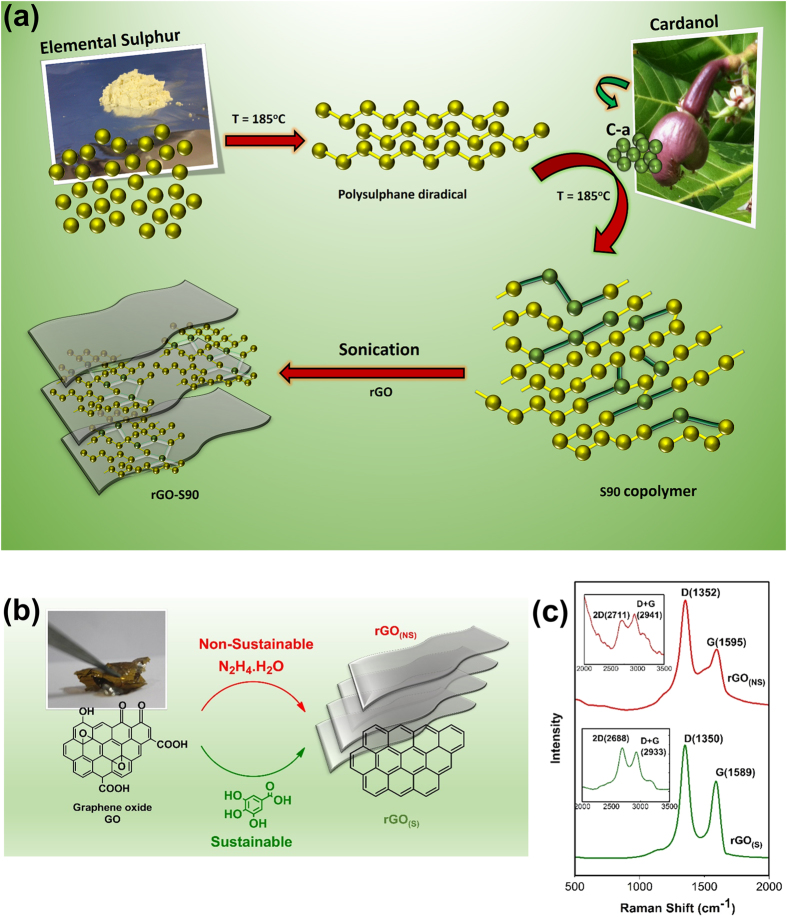
Synthetic schemes and characterization. (**a**) Schematic representations of S90 copolymer preparation. (**b**) Schematic representations of reduced graphene oxide preparation via non-sustainable and sustainable routes. (**c**) Raman spectra of rGO_(NS)_ and r-GO_(S)_.

Figure 1a represents the synthetic scheme of cardanol benzoxazine monomer C-a and the preparation of S90 copolymer via inverse vulcanization route. The monomer C-a was synthesized by Mannich-like condensation reaction of aniline with cardanol. Elemental sulfur exhibited melting at ~130 °C followed by free radical ring opening polymerization to form linear polysulphane diradicals, which were trapped by C-a. The growing radicals reacted at the alkylene chain and benzoxazine ring^50^ of C-a to form S90 copolymer. Proton nuclear magnetic resonance (^1^H-NMR) spectrum of S90 copolymer (Figure S1) confirmed the absence of any unreacted C-a monomer due to disappearance of signals of benzoxazine ring and double bonds (4.5–5.5 ppm) and simultaneously appearance of signals due to methylene hydrogens in Mannich bridge Ar-CH_2_-N and N-CH_2_-S at 3.5–4.5 ppm indicative of copolymerization. In addition, a relative shift in signals and disappearance of protons in aliphatic region (2.5–3.0 ppm) was also observed. Raman spectroscopy (Figure S2) of S90 copolymer also showed the signatures 500–800 cm^−1^ and 2800–3100 cm^−1^ corresponding to S-S, C-S and C-H linkages confirming the formation of copolymer^51^. The stability of the S90 copolymer [weight average molecular weight of 5.3 × 10^3 ^g mol^−1^ and a polydispersity index (PDI) of 3.7] in solution was confirmed by performing gel permeation chromatography (GPC) analysis (Figure S3) at different periods of storage (0^th^ and 17^th^ day). The GPC traces clearly suggest the stability of the polymer architecture under solvated conditions. The S90 copolymer exhibited two melting endotherms at 104 and 116 °C (Figure S4) as determined using differential scanning calorimetry (DSC) suggesting the existence of different crystalline domains in the polymer architecture.

GO was prepared using modified Hummer’s method which was then reduced to rGO using two alternative synthetic methodology, namely gallic acid, a natural occurring phenolic acid reductant and hydrazine hydrate (Fig. 1b) to form rGO_(S)_ and rGO_(NS)_ respectively. FTIR spectra of GO, rGO_(S)_ and rGO_(NS)_ are shown in Figure S5. The peaks centered at 3100, 1718, 1615, 1350, 1220, 1050 cm^−1^ in the FT-IR spectra of GO are attributed to hydroxyl, carboxyl, carbonyl, aromatic C = C, epoxy, ester stretching vibrations respectively. Both rGO_(S)_ and rGO_(NS)_ showed the absence of oxygenated functionalities peaks suggested successful reduction. The spectra of rGO_(S)_ showed no IR frequencies corresponding to gallic acid suggesting its absence and purity of the synthesized reduced graphene prepared via sustainable route. Raman spectra of rGO_(S)_ and rGO_(NS)_ (Fig. 1c) showed a strong band at 1589 and 1595 cm^−1^ (G band) corresponding to the first order scattering of the E_2g_ phonon mode of sp^2^ bonded C atoms and a weak band at 1348 and 1354 cm^−1^ (D band) arising from breathing mode of A_1g_ symmetry at K point. The I_D_/I_G_ intensity ratio was calculated as 1.42 rGO_(S)_ and 1.81 rGO_(NS)_ suggesting the development of more sp^2^ structural domains in the former with appreciable synthetic viability. In addition to D and G peaks, some other peaks are clearly visible at 2688/2711 (2D-mode) and 2933/2941 (D + G-mode) for rGO_(S)_/rGO_(NS)_ as shown in Fig. 1c (inset). In particular, the 2D and G bands represent the key features of Raman spectroscopy for the identification of graphene sheets. The 2D band peak at 2690 cm^−1^ is due to the highest optical branch phonons near the K point at the Brillouin zone boundary and the G band peak at 1575 cm^−1^ is due to the two-fold degenerate E_2g_mode at the C-point. The relative intensity ratio of I_2D_/I_G_ can be used to distinguish the number of layers of graphene sheets. The integral intensity ratio I_2D_/I_G_ of >2, 1–2, and <1 correspond to single-layered, double-layered and many-layered graphene, respectively. With increasing number of layers, the intensity ratio of I_2D_/I_G_ decreases whilst the FWHMs of the 2D peaks increases. For a good comparison, the spectra were normalized to give approximately the same relative intensity for all the samples. The I_2D_/I_G_ intensity ratio is less than 1 verifying existence of many layered graphene sheet and no single-layered graphene sheet was identified by the Raman spectroscopy^52^.

**Figure 2:**
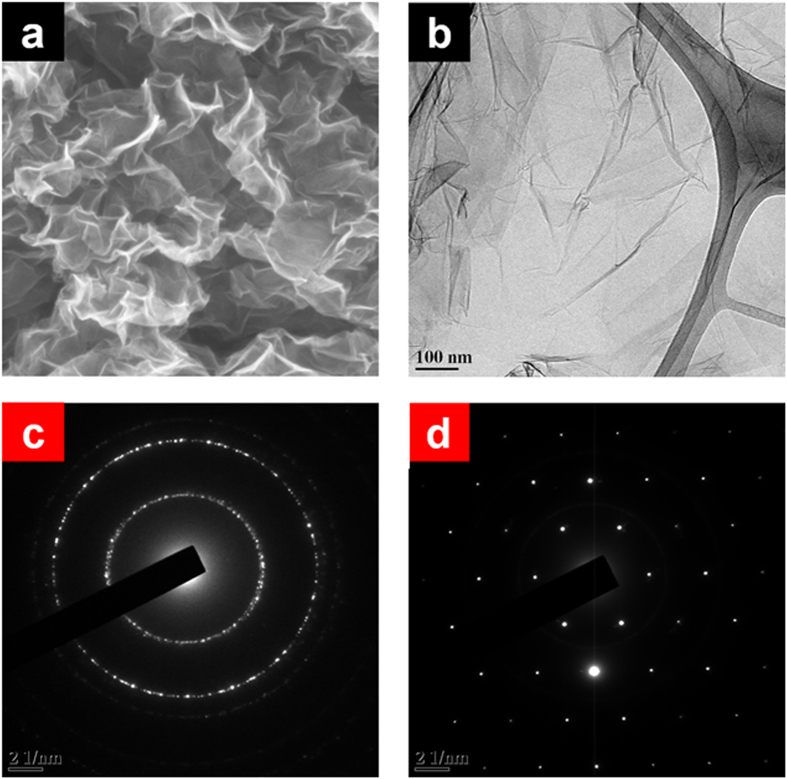
Microstructural analysis of rGO_(S)_. (**a**) SEM image and (**b**) TEM image of rGO_(S)_. (**c**) SAED pattern of graphene oxide (GO). (**d**) SAED pattern of rGO_(S)_.

**Figure 3:**
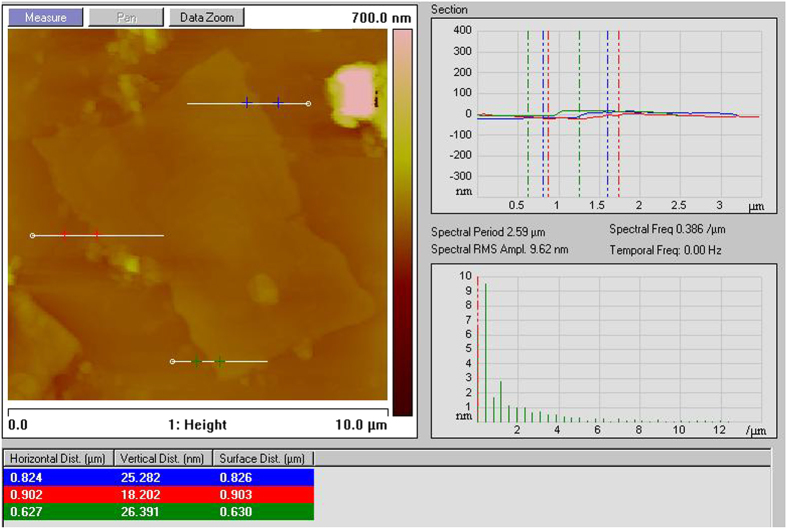
AFM characterization. AFM images of rGO_(S)_.

To understand the microstructural properties of bulk rGO prepared via sustainable route (*i.e.*, rGO_(S)_) scanning electron microscopic (SEM) as well as transmission electron microscopic (TEM) images were taken. Figure 2 represented the morphological features of rGO prepared via sustainable route. Both SEM (Fig. 2a) and TEM (Fig. 2b) images, showed the presence of wrinkles, ripples and scrolls in the rGO_(S)_ suggesting the occurrence of few-layered graphene sheets. Furthermore, selected area electron diffraction (SAED) patterns of GO and rGO_(S)_ (Fig. 2c,d) were compared to understand the successful reduction. A diffused diffraction ring pattern typical of a disordered structure is observed in GO illustrating the disruption of conjugated structure of graphite due to chemical oxidation. However, SAED image of rGO_(S)_ showed set of six-fold symmetric diffraction points of a typical hexagonal configuration^53^. This illustrates the formation of crystalline states in rGO_(S)_ representing the restoration of aromatic graphene structure and successful chemical reduction of GO using gallic acid. The thickness of the rGO_(S)_ was determined using atomic force microscopy (AFM) as shown in Fig. 3. The as-synthesized rGO_(S)_ consisted mainly of a corrugated surface with a height range between 18–26 nm nanometers and a lateral size of several micrometers. AFM analysis identified the absence of single-layered graphene (less than 1 nm) domains^53^. The existence of multi-layered graphene architecture in rGO_(S)_ was also confirmed by TEM, SEM and Raman analysis.

**Figure 4:**
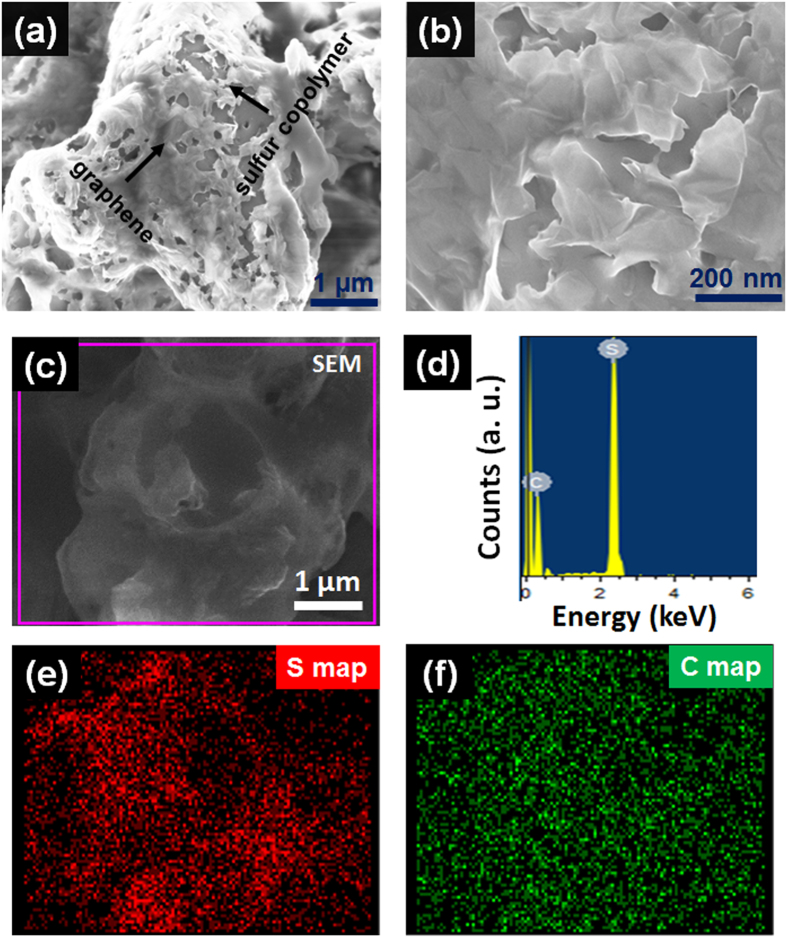
Microstructural analysis of S90- rGO_(S)_ composite. SEM images (**a**) at low magnification and (**b**) at higher magnification. (**c**,**d**) SEM image and EDX spectrum captured for the selected region. (**e**,**f**) Sulfur and carbon mapping of S90-rGO_(S)_ composite at the selected area.

Figure 4a shows SEM image of S90-2.5% rGO_(S)_ composite. A zoom-in SEM image (Fig. 4b) shows the uniform wrapping of rGO_(S)_ on sulfur copolymer surface. To verify the composition of our S90-2.5% rGO_(S)_ composite energy dispersive X-ray spectroscopic (EDX) characterization and elemental mapping of our material were carried out. From EDX spectra and elemental mapping of S90-2.5% rGO_(S)_ (Fig. 4c–f), uniform distribution of sulfur atoms throughout S90 copolymer and conformal wrapping of graphene sheets on the copolymer were confirmed.

**Figure 5:**
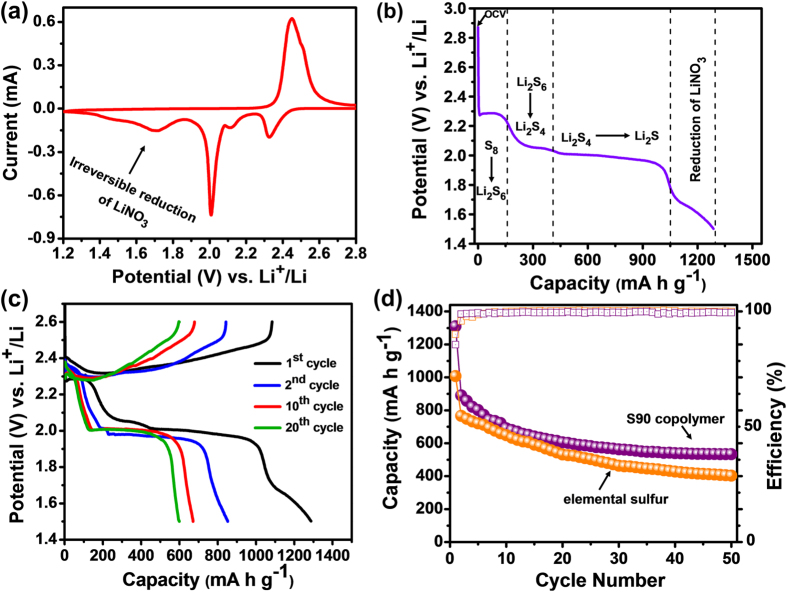
Electrochemical characterizations of bulk S90 copolymer. (**a**) Cyclic voltammetry (1^st^ cycle) of S90 copolymer at a scan rate of 20 μV/s and within the potential range 1.2–3.0 V. (**b**) Illustration of first discharge profile for S90 cathode at 200 mA g^−1^. (**c**) Charge-discharge profiles of S90 cathode at 200 mA g^−1^. (**d**) Charge-discharge cycling of S90 electrode at 200 mA g^−1^.

The electrochemical characterizations of S90 copolymer were evaluated using 2016 coin cells with lithium foil as anode. Figure 5a, represents the low scan rate (20 μV s^−1^) CV curve of bulk S90 copolymer cathode for the first cycle showing three reduction peaks and one oxidation peak. The reduction peak at 2.35–2.4 V is attributed to the conversion of sulfur to higher order linear polysulfides. The peak at 2.0–2.05 V is related to the further reduction of higher order polysulfides to lower order polysulfides, indicating multiple reaction feasibility of sulfur with lithium ions. The third peak in the potential range of 1.65–1.7 V is attributed to the irreversible reduction of LiNO_3_ additive in the electrolyte^54^. In addition, only one oxidation peak was observed at 2.3–2.35 V in the subsequent charge process, corresponding to the transformation of lower ordered polysulfides to higher ordered polysulfides. To study the lithium ion storage capacity of the bulk 90 copolymer, charge/discharge performances were performed within the potential window of 1.5–2.6 V at a current density of 200 mA g^−1^. Figure 5b illustrates the initial discharge profile of the S90 cathode at 200 mA g^−1^. As it is obvious that the positions of charge/discharge plateaus exactly corresponds to the peaks obtained during cyclic voltammetry, the initial discharge profile also exhibits three discharge plateaus at around 2.28, 2.0 and 1.7 V, respectively. Figure 5c demonstrates the charge-discharge profiles corresponding to 1^st^, 2^nd^, 10^th^ and 20^th^ cycles of electrode-A at 200 mA g^−1^ rate. Figure 5d shows the cycling performance of S90 cathode at the current density of 200 mA g^−1^. It is notable that there is a huge capacity loss at the 2^nd^ cycle. This extra capacity during the first discharge of the electrodes comes from the irreversible reduction of LiNO_3_ at the potential range of 1.5–1.7 V to form solid electrolyte interphase (SEI), resulting in low coulombic efficiency of the first cycle (ca. 85%). However, from 2^nd^ cycle onwards the coulombic efficiency was >98.5%. S90 electrode exhibited a reversible discharge capacity of 892 mA h g^−1^ at 2^nd^ cycle which degrades rapidly in first 20 cycles and becomes stable after 30 cycles. After 50 cycles the S90 copolymer delivers a discharge capacity of 528 mA h g^−1^. The initial capacity fading for S90 copolymer could be explained based on the low electronic conductivity and polymeric nature of the sample that may block both electronic as well as ionic pathway resulting to the poor Li^+^ ions and electrons accessibility towards active sulfur in S90 copolymer. However, the S90 electrode exhibited better cycling stability compare to the electrode made from pure sulfur. The better cycling performance of S90 copolymer could be due to the chemical attachment of sulfur atoms/chains with organic part (*i.e.*, Bzc) which can reduce active sulfur dissolution into electrolyte. In addition, the “plasticizing effect” of lower ordered organosulfides which get formed along with lower order lithium polysulfides (Li_2_S_3_/Li_2_S_2_) at lower potential during initial discharge of sulfur copolymer^14^ could also be another reason for improved performances of S90 copolymer.

**Figure 6:**
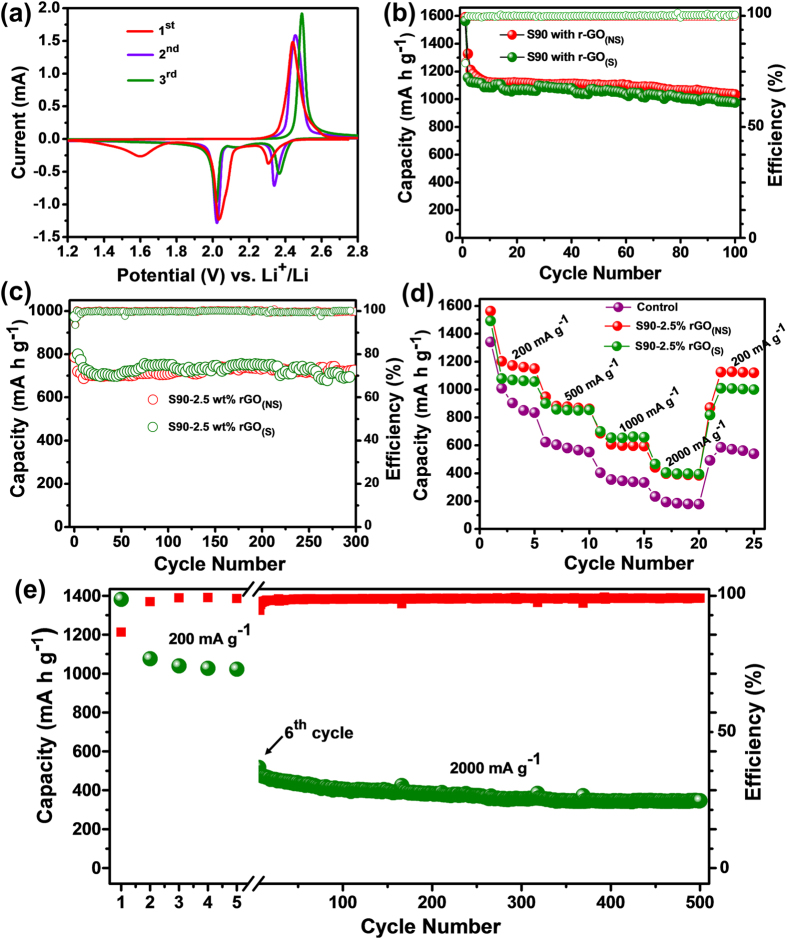
Electrochemical characterizations of S90-2.5% r-GO_(S)_ & S90-2.5% r-GO_(NS)_ composites. (**a**) Cyclic voltammetry of S90-2.5% r-GO_(S)_ at a scan rate of 20 μV/s and within the potential range 1.2–3.0 V. (**b**) Cycling performance comparison between both the S90-2.5% r-GO composites at 200 mA g^−1^ rate. (**c**) Cycling performance comparison between both the S90-2.5% r-GO composites at 1000 mA g^−1^ rate. (**d**) Rate performance comparison between both S90-2.5% r-GO composites and bulk S90 copolymer at different current rates. (**e**) Long term cycling performance of S90-2.5% r-GO_(S)_ composite at 2000 mA g^−1^ current rate.

However, to achieve better electrochemical performances from this copolymer, conductive fillers with excellent conductivity (like: carbon nanotubes or graphene) need to incorporate in the S90 copolymer before using as cathode active material. Graphene has been used as a conductive filler in this work due to its extremely high surface area and high electronic conductivity which can easily create even better percolation network throughout the copolymer with its minimum presence. A reduced graphene oxide was synthesized via green and sustainable route (rGO_(S)_) using gallic acid as reductant as well as stabilizer, and 2.5% (by mass) of it was dispersed into the copolymer. For comparison, the same amount of reduced graphene oxide synthesized though “non-sustainable” hydrazine process (rGO_(NS)_) was dispersed into the S90 copolymer and used as cathode material for Li-S batteries. Figure 6a displays the CV curves for S90-2.5% rGO_(S)_ composite electrode. The almost equal area of first five curves certifies the excellent electrochemical stability and reversibility of the S90-2.5% rGO_(S)_ composite. The S90-2.5% rGO_(S)_ composite exhibited a reversible capacity of 1156 mA h g^−1^ at 2^nd^ cycle at 200 mA g^−1^ rate (Fig. 6b). In terms of active material utilization rate at the 2^nd^ cycle, the S90-2.5% rGO_(S)_ composite had a higher utilization rate of 69.14% (1156 mA h g^−1^) of theoretical value than that of bulk S90 copolymer at 53.35% (892 mA h g^−1^). A reversible capacity up to 975 mA h g^−1^ was retained after 100 cycles, which is much higher than that of conventional sulfur-2D graphene composites (100^th^ cycle capacity = 910 mA h g^−1^, current rate = 167.2 mA g^−1^, sulfur loading = 40%^55^ or 100^th^ cycle capacity = 476 mA h g^−1^, current rate = 167.2 mA g^−1^, sulfur loading = 57.5%^56^), sulfur-3D graphene composites (100^th^ cycle capacity = 700 mA h g^−1^, current rate = 167.2 mA g^−1^, sulfur loading = 73%^57^) and the cathode made of electrodeposited sulfur onto graphene sheets (900 mA h g^−1^ after 60 cycles at 100 mA g^−1^, sulfur loading = 70.2%^58^). The superior performances of the S90-2.5% rGO_(S)_ composite material in terms of improved specific capacity and improved capacity retention is presumably caused by the use of high electronic conductivity and high surface area of rGO_(S)_ which induces an improved percolation network throughout S90 copolymer, facilitating Li^+^ ion transport through the electrode, as a result of which Li^+^ ions accessibility towards active sulfur increases. However, from Fig. 6b it is visible that S90-2.5% rGO_(S)_ composite exhibited almost same performance as S90-2.5% rGO_(NS)_ composite over 100 cycles, indicating that the similar type of percolation network as S90-2.5% rGO_(NS)_ composite was achieved for S90-2.5% rGO_(S)_ composite. Figure 6c, represents the cycling performances of S90-2.5% rGO_(S)_ and S90-2.5% rGO_(NS)_ composites at 1000 mA g^−1^ current rate. Both the composites exhibited an initial reversible capacity around 810 mA h g^−1^. A reversible capacity up to 700 mA h g^−1^ (86.42% of initial capacity) was retained after 300 cycles. It can be observed from Fig. 6d that the rate capability of S90 copolymer was also increased upon graphene incorporation. The corresponding charge-discharge profiles of S90-2.5% rGO_(S)_ at different current rates are shown in Figure S6. From Fig. 6d, it can be observed that S90-2.5% rGO_(S)_ composite exhibited almost similar rate performance as S90-2.5% rGO_(NS)_. However, to gain insight into the reason why both S90-2.5% rGO_(S)_ and S90-2.5% rGO_(NS)_ composites exhibited such similar electrochemical performances, the kinetics of both S90-2.5% rGO_(S)_ and S90-2.5% rGO_(NS)_ composite cathodes have been investigated through *in-situ* electrochemical impedance spectroscopy (EIS) measurements. The electrochemical impedance spectroscopy results for both the cells cycled over 100 cycles were compared. Generally, an EIS spectra is composed of a high-frequency semicircle related to the charge transfer process and low frequency straight line corresponding to a semi-infinite Warburg diffusion process. Both the composites exhibited almost identical sized semi-circle at low frequency region (Figure S7), indicating almost same amount of charge transfer resistance (R_ct_), as expected. The long term cycling performance of S90-2.5% rGO_(S)_ composite at 2000 mA g^−1^ current rate after the initial five cycles of activation at 200 mA g^−1^ is shown in Fig. 6e.

**Figure 7:**
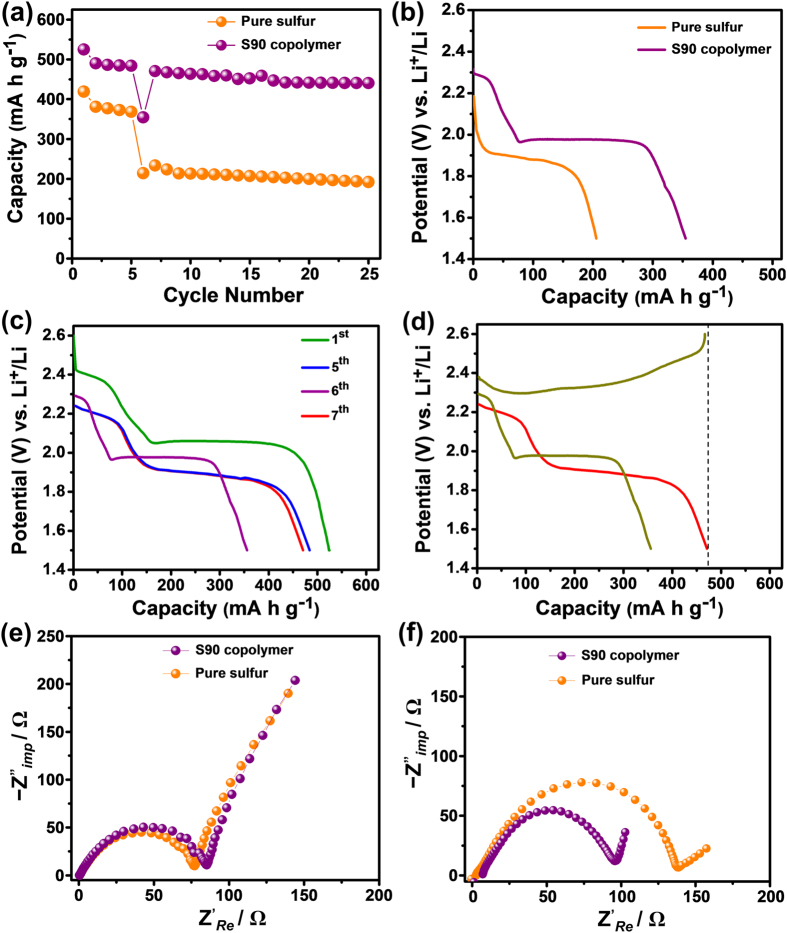
Self-discharge studies. (**a**) Cycling performances (at 500 mA g^−1^ current density) of pure sulfur and bulk S90 copolymer cathodes with 120 h rest time. (**b**) discharge profiles of pure sulfur and S90 electrode after the rest time of 120 h. (**c**) discharge profiles of S90 copolymer electrode for 1st, 5th (before rest), 6th (after rest), and 7th cycles. (**d**) charge-discharge profile of S90 cathode for 6th (after rest) discharge, 6th charge and 7th discharge. (**e**) EIS measurements of fresh cells composed of pure sulfur and S90 electrodes at OCV. (**f**) EIS measurements of both the cells after the rest time of 120 h.

Considering the view point from commercialization of Li-S battery in future, low self-discharge behavior (or long shelf-life behavior) of the cell is necessary. Most commonly, LiNO_3_ is used as an electrolyte additive to protect the lithium anode (by forming a stable solid electrolyte interface) from higher order polysulfides and dissolved sulfur molecules, thus preventing the self-discharge of Li-S batteries^59^. However, to investigate the answer of the question, “can the active sulfur dissolution in Li-S battery be suppressed through chemical confinement strategy?”, self-discharge studies have been carried out for both S90 copolymer and elemental sulfur cathode using an electrolyte containing no LiNO_3_ additive. Initially, both the cells were cycled for 5 cycles and then kept in rest for 120 h. The cells were charged to 2.6 V before keeping in rest. After 120 h of rest time, the cells were discharged as usual and recharged to 2.6 V. Then they were further cycled for another 20 cycles. From Fig. 7a, it can be observed that both the electrodes showed stable cycling performances for initial five cycles. During rest period the open circuit voltage (OCV) gradually decreases for both the cells. After 120 h rest time, OCV became very stable at 2.33 V for S90 copolymer cell, while for pure sulfur cell it was 2.20 V (Fig. 7b). After 120 h rest time, the S90 copolymer cathode exhibited two distinct discharge plateaus, while the pure sulfur cathode showed only lower discharge plateau (since the OCV for pure sulfur cathode was less than the potential at which the reactions corresponding to upper discharge plateau usually take place). This observation manifests that the self-discharge rate for S90 copolymer electrode was significantly lower compared to elemental sulfur cathode. The rapid fall in OCV and disappearance of upper discharge plateau for pure sulfur cathode can be related to the formation of lower order polysulfides (*i.e.*, Li_2_S_3_/Li_2_S_2_) from the reaction between rapidly dissolved unbound sulfur and bare lithium^60^. In brief, the dissolved S_8_ molecules migrate towards anode and chemically reacts with unprotected lithium metal to produce initial discharge product Li_2_S_8_. This unstable Li_2_S_8_ further reacts with lithium to form more stable lower order polysulfides. On other hand, due to chemical attachment of sulfur with the organic part in S90 copolymer, the dissolution of sulfur for S90 cathode was less compare to pure sulfur cathode. Figure 7c shows the discharge profiles of S90 copolymer electrode for 1^st^, 5^th^ (before rest), 6^th^ (after rest), and 7^th^ cycles. From Fig. 7c, it is noteworthy to mention that there was 98.6% retention of 5^th^ cycle (the cycle before rest time) discharge capacity (458 mA h g^−1^) at 7^th^ cycle (the cycle after rest time, discharge capacity = 452 mA h g^−1^) for S90 cathode. Moreover, Fig. 7d reveals that for S90 electrode the 6^th^ cycle charge capacity was very much higher (447 mA h g^−1^, almost same as next cycle discharge capacity) as compare to the discharge capacity of the same cycle (the discharge after rest time). To investigate the reason behind these observations, electrochemical impedance measurements were taken for both pure sulfur and bulk S90 copolymer electrodes at different stages. Both the electrodes made of pure sulfur and S90 copolymer, exhibited almost same charge transfer resistance (R_ct_) at their open circuit voltage (OCV) before charge-discharge was performed (Fig. 7e). A remarkable difference in R_ct_ was observed when EIS were taken for both the cells after 120 h of rest time (Fig. 7f). The S90 cathode showed very much lower charge transfer resistance (R_ct_ = 97 Ω) than pure sulfur cathode (R_ct_ = 135 Ω). The lower charge transfer resistances for S90 electrode after 120 h of rest time could be due to the presence of organosulfur units in S90 copolymer. During initial electrochemical discharge, the organosulfur units in the copolymer get reduced to form lower order lithium polysulfides (Li_2_S_2_/Li_2_S) and lithium organosulfides^22^. These lithium organosulfides then co-deposited within the matrix of insoluble/insulating lower order lithium polysulfides and react to form higher order lithium organosulfides which is soluble into electrolyte. Thus the organosulfur units present in copolymer acts as a “plasticizers” in the insoluble/insulating Li_2_S/Li_2_S discharge product phase, preventing their irreversible deposition on electrode surfaces (during cycling or during rest time) to reduce cell resistance as well as capacity fading. To interrogate the “plasticizing effect” of organosulfur units on the deposition of Li_2_S_3_/Li_2_S_2_ on the electrode surfaces, morphological characterizations of the electrodes after cycling (at fully charged state) were also conducted. From Figure S8, dense discharge products (Li_2_S_3_/Li_2_S_2_) could be observed on the surface of elemental sulfur electrode. These insoluble and insulating products, formed from deep discharge of elemental sulfur, “paste” on sulfur electrode which not only lead to the blockage of ionic/electronic transport, but also lead to loss of active material, both of which could result in the capacity fading. In contrast, the S90 copolymer electrode showed no such type of insulating product deposition. Hence the regeneration of capacity was observed at 6^th^ charge (Fig. 7d) for S90 copolymer while for elemental sulfur no such capacity retention was observed after 6^th^ cycle of charge-discharge. Therefore, the presence of organosulfur units in the sulfur copolymer could significantly suppress the irreversible deposition of insulated end discharge products on the electrode surface during cycling as well as during the rest time.

In summary, a novel sulfur-rich (90% sulfur) copolymer has been synthesized via a facile, sustainable and scalable process utilizing two industrial waste- sulfur, a petroleum waste and cardanol, an agro waste. One of the monomers, cardanol benzoxazine (C-a, the organic part of copolymer) was extracted from cardanol and synthesized by a solventless, one pot and atom economized process with water as the only by-product of the reaction. The solubility of the copolymer allowed structural characterization using solution-based techniques. We demonstrate that the chemical attachment of sulfur with the organic part (C-a) and the presence of organosulfur units in this copolymer can significantly compensate the negative aspects of elemental sulfur to improve the Li-S battery performances without the need of any nanoscopic synthesis. However, the large structural property of the organic part in the copolymer impeded it to perform the best electrochemical activities. Therefore, in addition reduced graphene oxide (rGO) has also been prepared in a green, scalable route utilizing a naturally occurring compound, gallic acid and used as conductive agent to get the better electrochemical performances. Finally, considering the synthetic approaches for sulfur copolymer and reduced graphene oxide and the intriguing features of their composite cathode in Li-S battery, we believe that this work can provide a valuable insight in the utilization of wastes as energy storage materials and the usage of sustainable carbon based nanomaterial as a conductive additive without compromising on cost or performance.

## Methods

### Materials

Cardanol was procured from Satya Cashew Chemicals Pvt. Ltd. (India), paraformaldehyde, sulfuric acid, sodium nitrate, potassium permanganate, hydrogen peroxide, hydrazine hydrate, aniline and liquor ammonia from Fisher scientific, gallic acid from Sigma Aldrich, chloroform from Finar, anhydrous sodium sulphate from Chemlabs, graphite flakes and sulfur (S_8_, 325 mesh, reagent grade) from Alfa Aesar. All the reagents were used as received.

### Synthesis of cardanol benzoxazine (C-a)

Cardanol benzoxazine was prepared from cardanol as per the procedure reported previously^39^. A mixture of cardanol (100 g, 0.33 mol), paraformaldehyde (19.8 g, 0.66 mol), aniline (30.1 mL, 0.33 mol) was gradually heated from room temperature to 70 °C then at 80 °C for 1 h followed by heating at 90 °C for 2 h. After cooling, water (500 mL) was added and organic layer was extracted with chloroform (2 × 100 mL). The organic layers were combined and dried over sodium sulphate and filtered. The solvent was removed under vacuo to give cardanol benzoxazine (C-a) in quantitative yield as red brown oil. v (film, ATR)/cm^−1^ 3008, 2926, 2854,1626, 1595, 1579, 1350, 1257, 1240, 1116, 1030, 995, 950 and 627 cm^−1^; ^1^H-NMR (500 MHz, CDCl_3_, δ ppm): 0.94 (CH_3_, t), 1.35 [(CH_2_)_n_, m], 1.85, 2.07 (CH_2_CH=, m), 2.59 (CH_2_Ar, t), 2.89 [CH_2_(CH=)_2_, m], 4.57 (s, ArCH_2_N–), 5.05 (–CH = CH–, dd), 5.37 (m, CH=, CH_2_ = CH–, –OCH_2_N–, –HC = CH_2_), 6.60(ArH, s), 6.72(ArH, d), 6.93 (ArH, m), 7.10 (ArH, d), 7.27 (ArH, m); LCMS (ESI Interface-positive ions, HRMS): C-a adduct: 415.2403, 416.2435, 417.2468, 419.2984, 420.3023 (as expected theoretically).

### Synthesis of sulfur-rich copolymer, poly (sulfur-random-cardanol benzoxazine) (S90)

Elemental sulfur (1.8 g) was added to a 15 mL glass vial equipped with a magnetic stir bar and heated to a temperature of 185 °C in an oil bath equipped with thermostat to obtain a clear orange coloured viscous molten phase. Cardanol benzoxazine (C-a) (0.2 g) was then added directly to the molten sulfur via a syringe and this mixture was stirred further at the temperature of 185 °C for 10 minutes. Mixture was cooled to room temperature slowly and poly(S-*r*-C-a) (S90) was obtained in quantitative yields.

### Synthesis of Graphene Oxide (GO)

GO (Graphite oxide) was prepared by a modified Hummer’s method^61^. Briefly, graphite flakes (2.0 g) were added to concentrated sulfuric Acid (46.0 mL) and sodium Nitrate (1.0 g) while keeping the temperature at 0 °C with continuous stirring. This was followed by a gradual addition of potassium permanganate (6.0 g) while maintaining the temperature below 20 °C. Upon completion of addition, ice bath was removed and resultant suspension was stirred. The solution turned a brownish gray in 30 minutes after which distilled water (92.0 mL) was added to the paste. The solution was finally treated with warm water (280.0 mL) and hydrogen peroxide (3%) to give brown colored GO. Hence, the formed GO was washed with water (8 × 500 mL) to remove all the acidic content and dried under vacuum to obtain yellowish brown solid (1.2 g, 60%).

### Synthesis of reduced Graphene Oxide (rGO)

As prepared graphene oxide (GO) was reduced following a green method using gallic acid as reducing agent instead of hydrazine. GO (260 mg) was dispersed in deionized water (65 mL) by sonication. Liquor Ammonia (25%) was added to this aqueous dispersion to adjust the pH value of the mixture to 12. Gallic acid (2.607 g, 15.3 mmol) was then added to the mixture followed by stirring at 95 °C for 10 h in a nitrogen atmosphere. rGO was filtered and subjected to washing with deionized water followed by acetone. Hence, obtained product was dried and weighed to give as black solid (150 mg, 58%).

### Synthesis of S90-rGO composite

S90 (1.46 g) was well-dispersed in THF (30 mL) by ultra-sonication for 1 h. This was followed by addition of rGO (37.5 mg) and resulting suspension was sonicated for 2 h for efficient blending. The solvent was removed under *vacuo* to give a gray powder in quantitative yield.

### Characterizations

Fourier Transform Infrared (FT-IR) spectra were recorded on a *Nicolet iS5* spectrometer equipped with iD5-ATR accessory, in the range of 4000 to 400 cm^−1^. Raman spectra were obtained using a JobinYvon HR800 Raman Microscope at 514.5 nm. Bruker AC 500 MHz FT-NMR spectrometer was used to record the ^1^H-NMR of the samples in CDCl_3_ using tetramethylsilane as the internal standard. Agilent HRMS 6540 Series Q-TOF was used for mass spectrometry of C-a monomer. GPC was recorded on a Viscotek Model 305 TDAmax with refractive index detector. Samples were solvated in tetrahydrofuran (THF) solvent (4–5 mg mL^−1^) and filtered through 0.2 μm PTFE filter before injection. GPC system was calibrated with polystyrene standards and data was analyzed using Omnisec software. Calorimetric studies were performed on a Differential Scanning Calorimeter (DSC, TA instruments Q 20). For dynamic DSC scans, samples (10 ± 2 mg) were sealed in aluminum pans, and heated from 50 to 160 °C at 10 °C min^−1^ under nitrogen atmosphere. X-Ray Diffraction studies were performed on Rigaku SmartLab X-Ray Diffractometer, CuK_α_ radiation (λ = 1.5406 Å). The surface morphology of samples was studied using a Scanning Electron Microscope (Zeiss EVO MA15) under an acceleration voltage of 1 kV. Samples were mounted on aluminium stubs and sputter-coated with gold and palladium (10 nm) using a sputter coater (Quorum-SC7620) operating at 10–12 mA for 120 s. The morphology and microstructure of bulk rGO_(S)_ and S90-2.5% rGO_(S)_ composite were characterized through field emission scanning electron microscopy (FESEM, Carl Zeiss Ultra 55) and high resolution transmission electron microscopy (HRTEM, JEOL-2100F). Energy-dispersive X-ray spectroscopy (EDX) and elemental analysis were recorded on a Carl-Zeiss Ultra 55 FE-SEM with Oxford EDX system.

### Cells fabrication and electrochemical measurements

The S90 copolymer cathode was prepared by mixing S90 copolymer as active material, super C-65 carbon black as conductive additive and polyethylene oxide (PEO) as binder with an overall ratio of 7:2:1 (by wt.) in acetonitrile. After stirring, the slurry was blade cast onto carbon-coated aluminium foil and then dried at 60 °C for 48 h. Electrodes using S90 copolymer with 2.5% rGO_(S)_ and 2.5% rGO_(NS)_ composites were prepared separately in the same way. The electrodes were cut into disks with a diameter of 12 mm. The average sulfur loading densities on the electrodes were ~1.0 (for S90) and ~0.95 mg cm^−2^ (for S90-2.5% rGO composite) respectively. Coin-type (CR2016) cells were assembled in an argon-filled glovebox. Lithium metal was used as both reference and counter electrode. Borosilicate glass microfiber filters (Whatmann) was used as separator soaked with 40 μL of electrolyte composed of 0.25 M LiTFSI and 0.1 M LiNO_3_ in 1:1 (v/v) mixture 1,3-dioxolane (DOL) and 1,2-dimethoxy ethane (DME). Cyclic voltammetry was carried out at a scan rate of 20 μV s^−1^ within the potential window 1.2–3.0 V vs. Li^+^/Li using Biologic VMP-3. The Galvanostatic charge-discharge experiments were performed using an Arbin BT-2000 within the potential range 1.5–2.6 V. The capacities were evaluated based on the mass of sulfur present in the S90 copolymer (90% sulfur) and S90-2.5% rGO composites (87.75% sulfur). Electrochemical impedance spectroscopy (EIS) measurements were carried out using Bio-logic VMP-3 over the frequency range of 10 mHz to 1 M Hz and with a voltage amplitude of 5 mV. All electrochemical measurements were carried out at a constant temperature of 20 °C.

## Additional Information

**How to cite this article**: Ghosh, A. *et al*. Sustainable Sulfur-rich Copolymer/Graphene Composite as Lithium-Sulfur Battery Cathode with Excellent Electrochemical Performance. *Sci. Rep*. **6**, 25207; doi: 10.1038/srep25207 (2016).

## Supplementary Material

Supplementary Information
