# Supplementary material for: Sustainable Sulfur-rich Copolymer/Graphene Composite as Lithium-Sulfur Battery Cathode with Excellent Electrochemical Performance
Source: Sci Rep. 2016 Apr 28;6:25207. doi: 10.1038/srep25207 (PMC4848474; doi:10.1038/srep25207)
Supplement: Supplementary Information [file srep25207-s1.pdf]

Supporting information for

Sustainable Sulfur-rich Copolymer/Graphene Composite as Lithium-Sulfur Battery Cathode with Excellent Electrochemical Performance

Arnab Ghosh[1], Swapnil Shukla[2], Gaganpreet Singh Khosla[2], Bimlesh Lochab[2*]& Sagar Mitra[1*]

[1]Electrochemical Energy Storage Laboratory, Department of Energy Science and Engineering, Indian Institute of Technology, Bombay, Powai, Mumbai 400076, INDIA.

[2]Department of Chemistry, School of Natural Sciences, Shiv Nadar University, Gautam Budh Nagar,Uttar Pradesh 203207,INDIA.

Corresponding Authors[*]:

sagar.mitra@iitb.ac.in

bimlesh.lochab@snu.edu.in

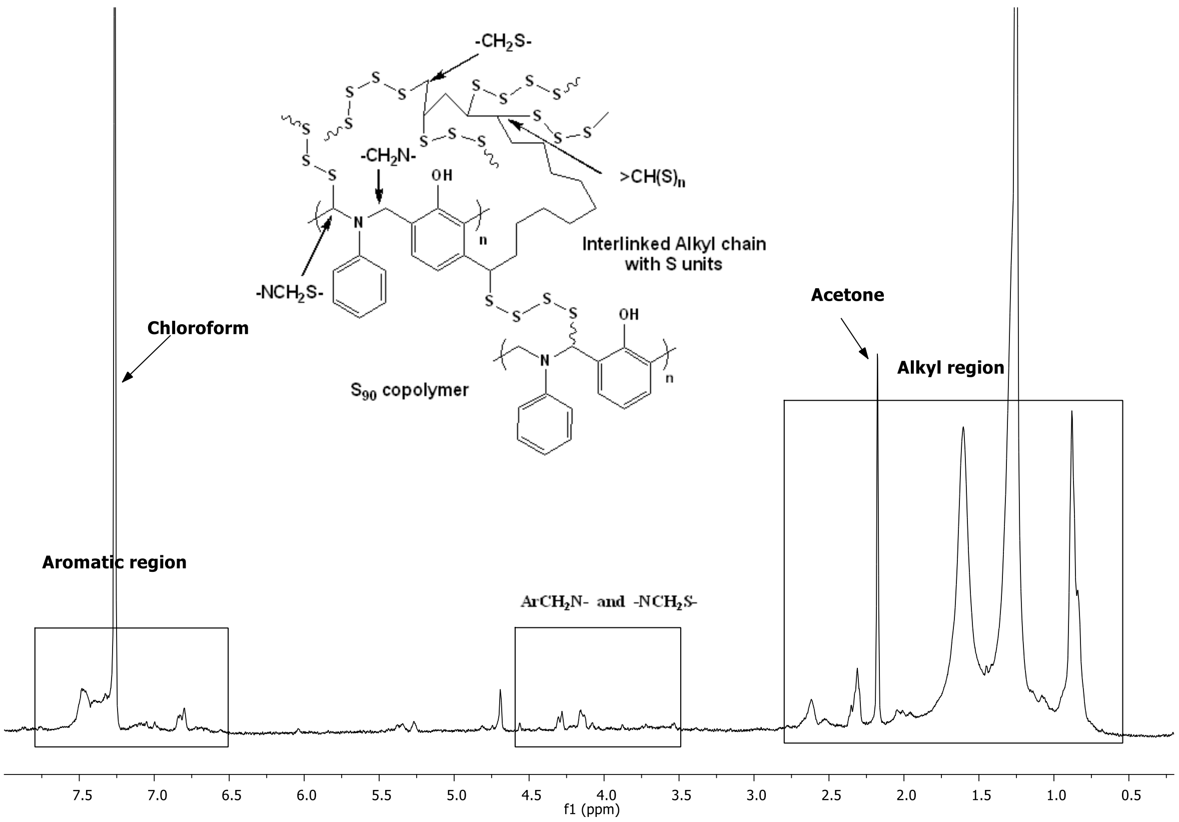

Figure S1. ^{1}H-NMR of S90 in $CDCl_3$ solvent.

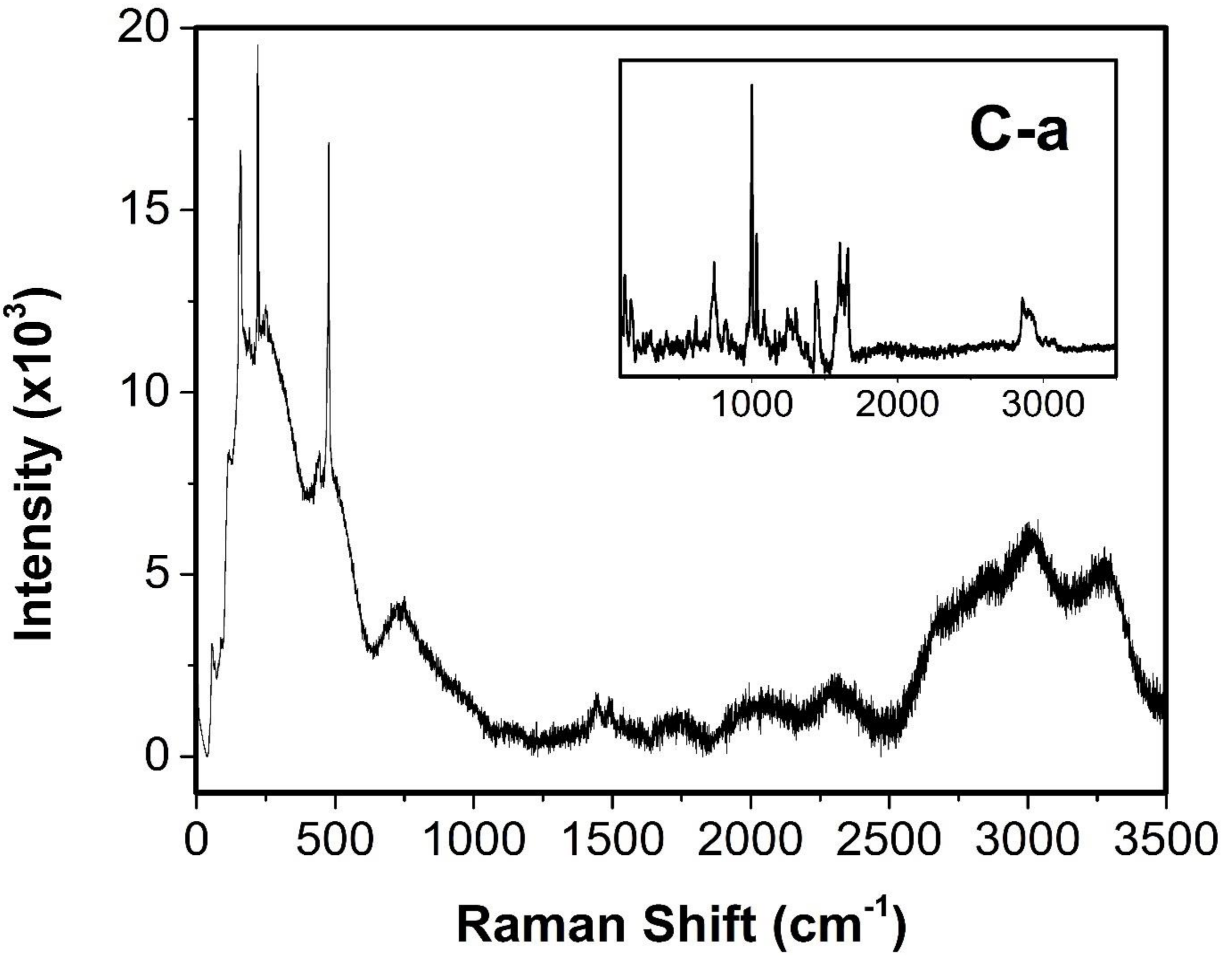

Figure S2. Raman spectra of S90; inset Raman spectra of C-a.

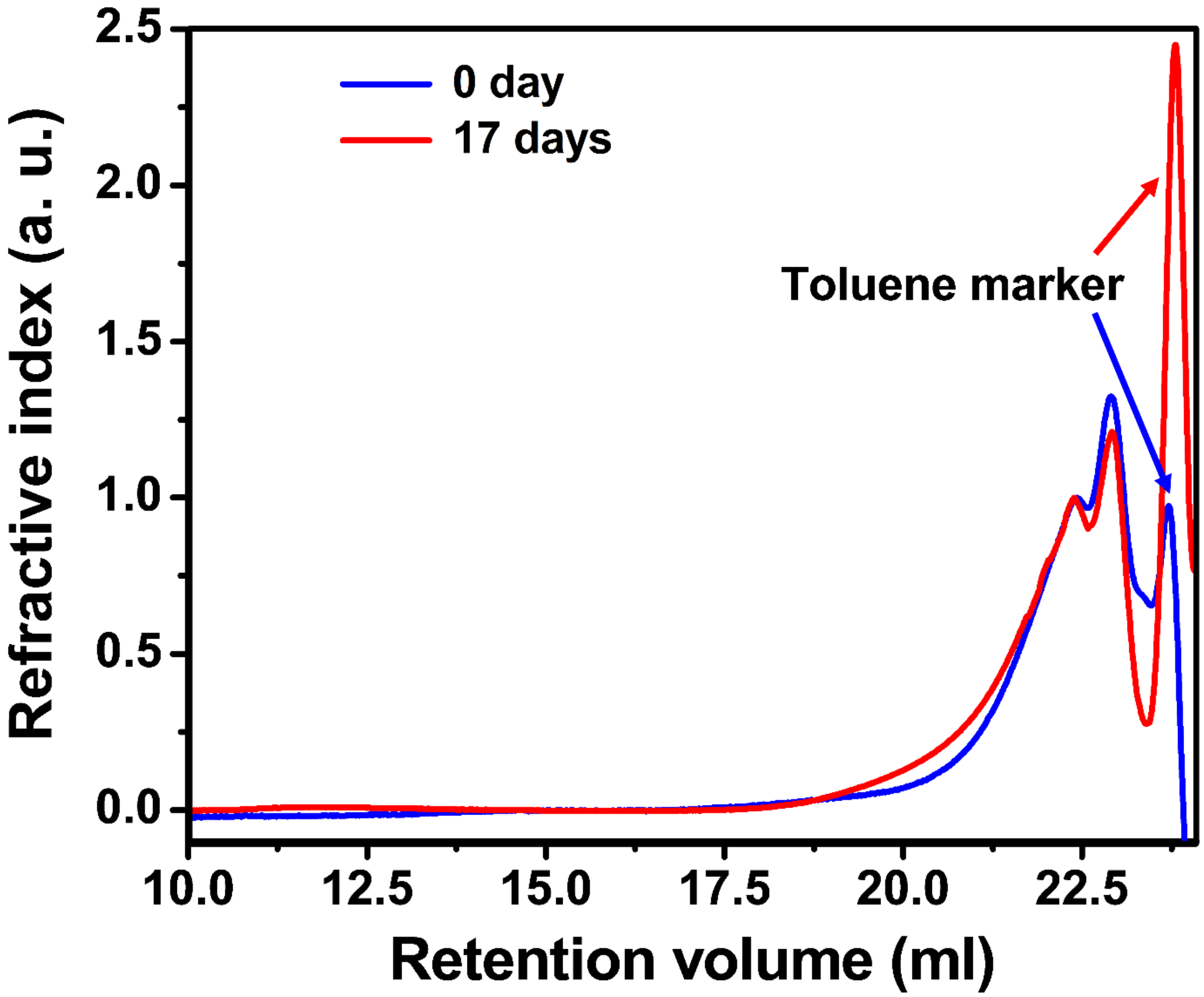

Figure S3. Normalised GPC trace of S90 copolymer at different period of storage.

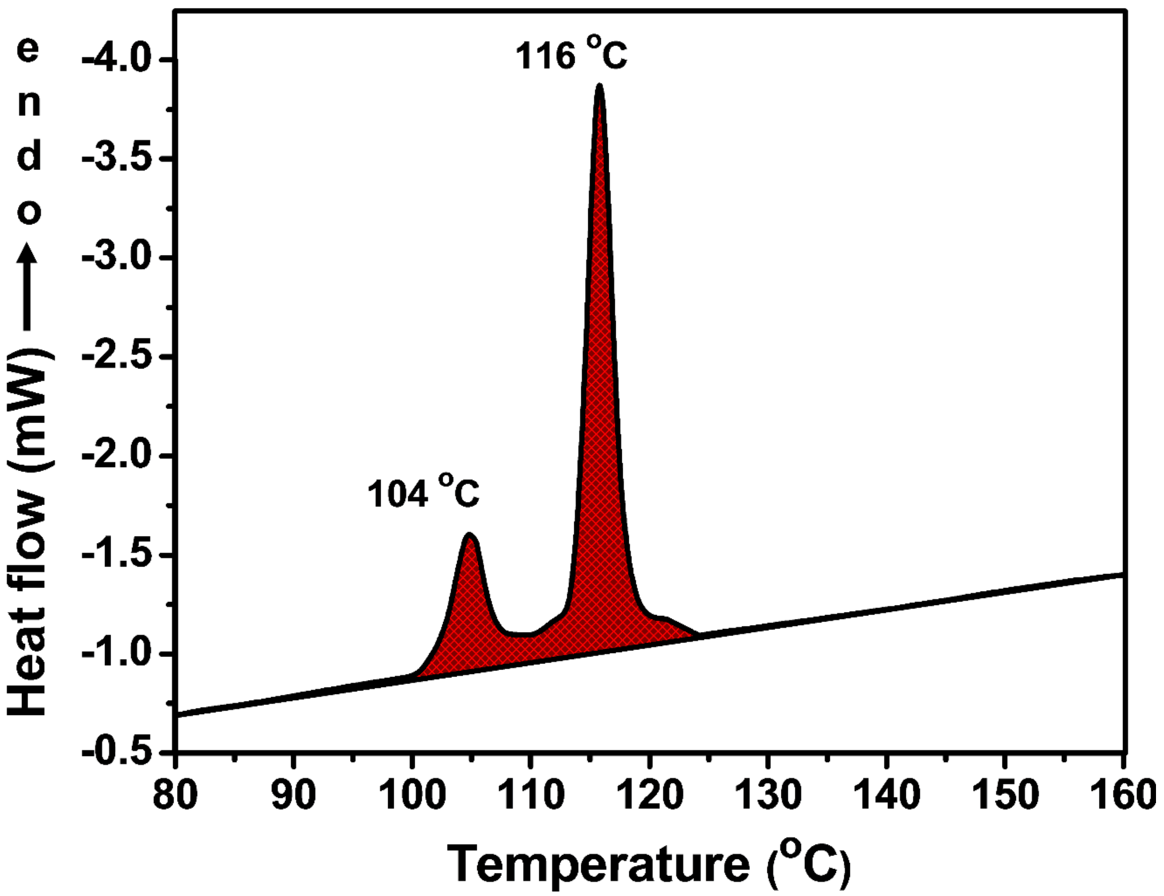

Figure S4. DSC trace of S90 copolymer.

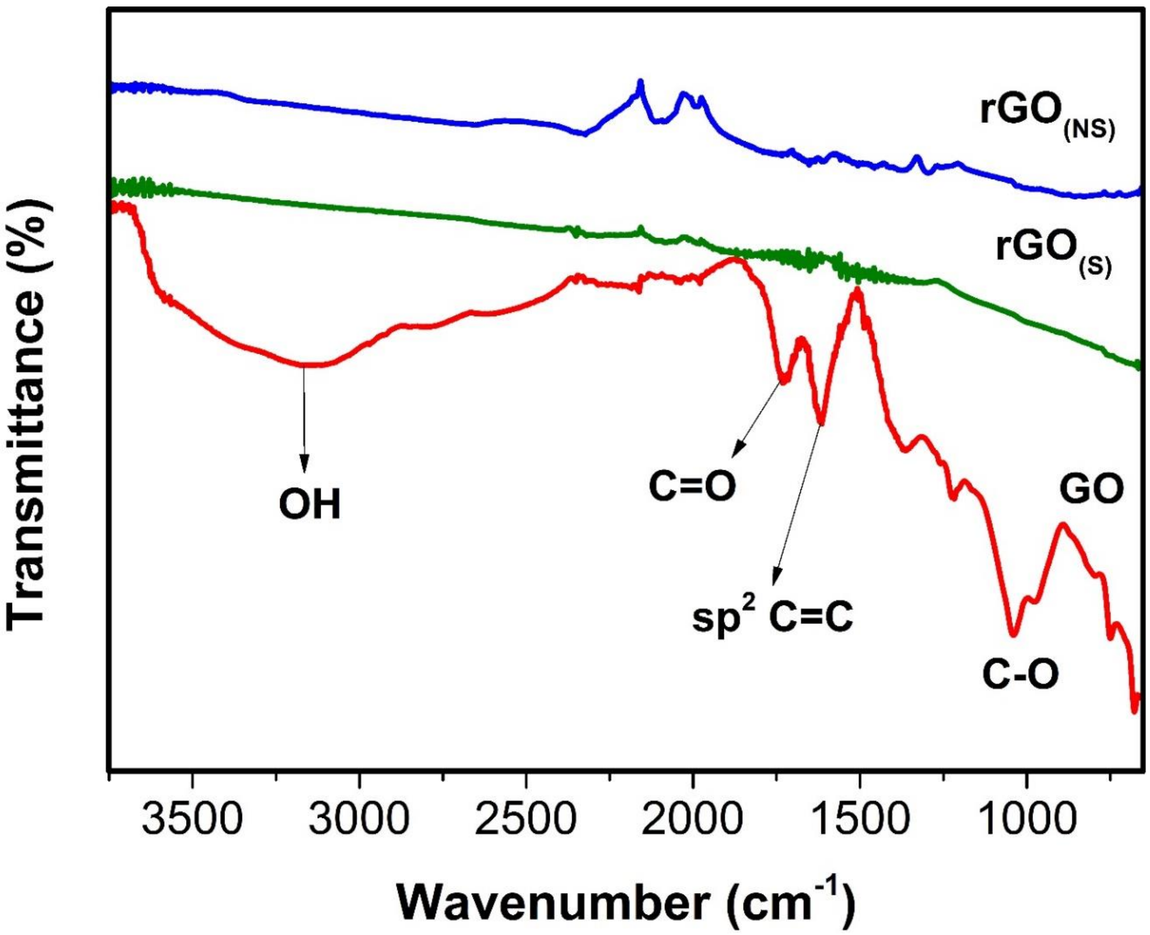

Figure S5. FTIR spectra of graphene oxide (GO), $rGO_{(S)}$ and $rGO_{(NS)}$.

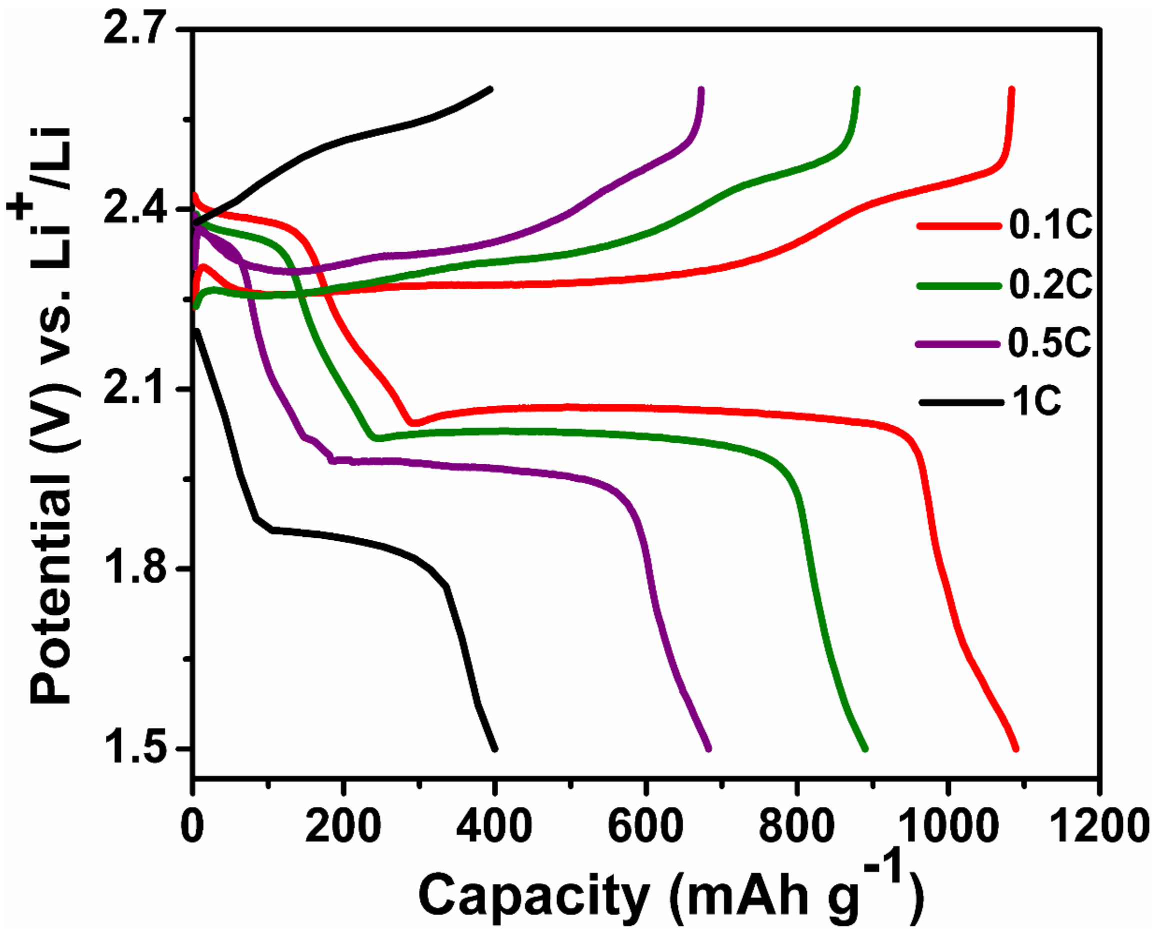

Figure S6. Charge-discharge profiles of S90-$rGO_{(S)}$ composite at different current rates.

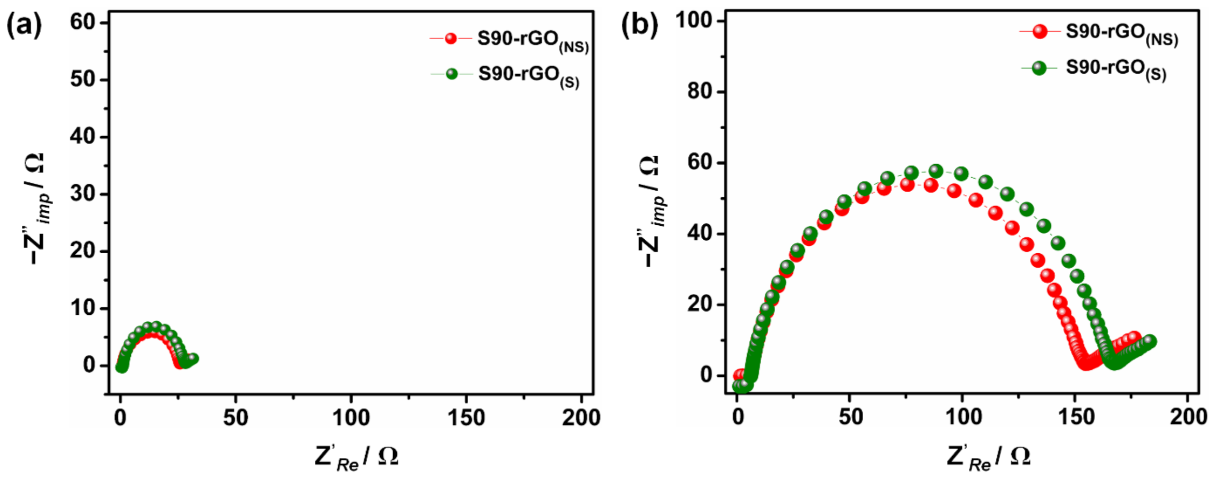

Figure S7. Nyquist plots of S90-2.5% $rGO_{(S)}$ and S90-2.5% $rGO_{(NS)}$ composites electrodes – (a) before cycling and (b) after 100 cycles (at fully charged state).

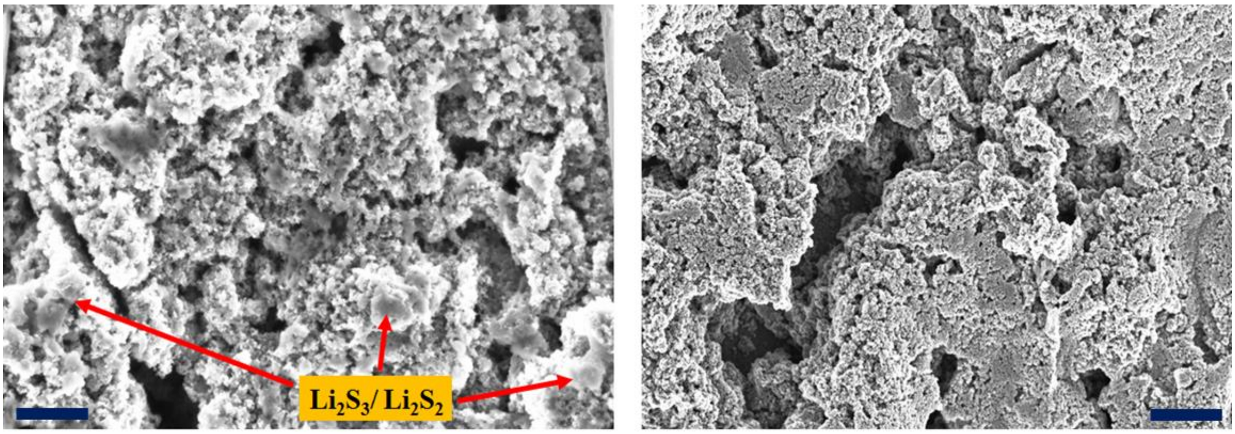

Figure S8. SEM images of elemental sulfur electrode (left) and S90 copolymer electrode (right) after 50 cycles at fully charged state (scale bar: 2 μm).

Materials	Sulfur Loading	Initial discharge capacity	Capacity loss per cycle	
			Cycles	Loss
Poly (S-r-Ca)/2.5 wt% rGOs *(This work)*	87.7 wt%	807 mA h g^{-1} (1000 mA g^{-1})	300	0.04 %
		502 mA h g^{-1} (2000 mA g^{-1})	500	0.06 %
Graphene/sulfur[1]	70 wt%	1000 mA h g^{-1} (1500 mA g^{-1})	300	0.1 %
Amino-functionalized reduced graphene oxide[2]	60 wt%	810 mA h g^{-1} (836 mA g^{-1})	300	0.057 %
Graphene wrapped N-doped double shelled hollow carbon sphere[3]	78 wt%	850 mA h g^{-1} (836 mA g^{-1})	200	0.19 %
Ultra-high surface-area hollow carbon nanosphere[4]	67 wt%	750 mA h g^{-1} (1672 mA g^{-1})	500	0.053 %
Sulfur infiltrated graphene based layered porous carbon[5]	68 wt%	900 mA h g^{-1} (836 mA g^{-1})	100	0.3 %
Coaxial graphene wrapped sulfur-coated carbon nanofibers[6]	50 wt%	615 mA h g^{-1} (1672 mA g^{-1})	1500	0.043 %
Hollow carbon nanofibers filled with MnO_2 nanosheets[7]	71%	915 mA h g^{-1} (836 mA g^{-1})	300	0.08 %

Table 1: Comparison of our sulfur copolymer cathode with previously reported sulfur cathode material structures in terms of specific capacity and cycling stability.

References

S1. Zhou, G. *et al. Adv. Mater.* **26**, 625-631 (2014).

S2. Wang, Z. *et al. Nat. Commun.* **5**, 5002 (2014).

S3. Zhou, G., Zhao, Y. & Manthiram, A. *Adv. Energy Mater.* **5**, 1402263 (2015).

S4. Xu, F. *et al. Nat. Commun.***6**, 7221 (2015).

S5. Yang, X., Zhang, L., Zhang, F., Huang, Y. & Chen, Y. *ACS Nano* **8**, 5208-5215 (2014).

S6. Lu, S., Cheng, Y., Wu, X. & Liu, J. *Nano Lett.* **13**, 2485-2489 (2013).

S7. Li, Z., Zhang, J. & Lou, X.W. *Angew. Chem. Int. Ed.* **127**, 13078-13082 (2015).
